# Undetected Omicron Transmission in Romania—Report of the First Detected Case of Locally Acquired Omicron Infection and Complete Epidemiological Investigation

**DOI:** 10.3390/diagnostics12020348

**Published:** 2022-01-29

**Authors:** Anca Streinu-Cercel, Oana Săndulescu, Victor Daniel Miron, Simona Paraschiv, Corina Casangiu, Robert Hohan, Leontina Bănică, Marius Surleac, Adrian Streinu-Cercel

**Affiliations:** 1 Carol Davila University of Medicine and Pharmacy, 050474 Bucharest, Romania; anca_sc@yahoo.com (A.S.-C.); oana.sandulescu@umfcd.ro (O.S.); simona.paraschiv@umfcd.ro (S.P.); astreinucercel@yahoo.com (A.S.-C.); 2 National Institute for Infectious Diseases “Prof. Dr. Matei Bals”, 021105 Bucharest, Romania; corina.casangiu@gmail.com (C.C.); robert.hohan@gmail.com (R.H.); leo_mirela@yahoo.com (L.B.); marius.surleac@gmail.com (M.S.); 3 Faculty of Biology, University of Bucharest, 077120 Bucharest, Romania

**Keywords:** SARS-CoV-2, omicron, variant of concern, epidemiological surveillance

## Abstract

The occurrence of the omicron variant of severe acute respiratory syndrome coronavirus 2 (SARS-CoV-2) has importantly impacted surveillance and diagnosis, and has changed the therapeutic landscape of coronavirus disease 2019 (COVID-19). We present the first documented case of locally acquired SARS-CoV-2 omicron variant in Romania in a patient with no recent travel outside the country. We also present the full results of the epidemiological investigation that led to the identification of the index case in a co-worker who had developed mild symptoms shortly after returning from the UK and who had undergone multiple rapid antigen tests with negative results prior to being tested by RT-PCR. We highlight potential lessons learned and describe further directions for actionable research and development in the field of COVID-19.

The occurrence of the omicron variant of severe acute respiratory syndrome coronavirus 2 (SARS-CoV-2) has importantly impacted surveillance and diagnosis and has changed the therapeutic landscape of coronavirus disease 2019 (COVID-19), compelling the medical world to rethink public health measures as well as prophylaxis and therapeutic decisions tailored to the particularities of this new variant of concern (VOC) [1].

We present the first documented case of locally acquired SARS-CoV-2 omicron variant in Romania in a patient with no recent travel outside the country. We also present the full results of the epidemiological investigation that led to the identification of the index case, and we highlight potential lessons learned and describe further directions for actionable research and development in the field of COVID-19.

On the evening of 9 December 2021, a 28-year-old female patient (Case 1) from Bucharest, Romania, experienced nasal congestion and otalgia and on the morning of 10 December 2021 she presented to the National Institute for Infectious Diseases “Prof. Dr. Matei Balș”, Bucharest, Romania where she was tested by rapid antigen test and RT-PCR for SARS-CoV-2. Both results came back positive, and she self-isolated at home starting the same day where she continued to be monitored. The patient had no history of travel outside the country in the past 14 days, but as part of the Institute’s epidemiological surveillance, she was also tested for VOC omicron and delta screening mutations (501Y for omicron and 681R for delta). The sample was found positive for the 501Y mutation and therefore whole genome sequencing was further performed to fully characterize the viral genome. Next-generation sequencing coupled with bioinformatic analysis confirmed that the viral strain was indeed omicron variant B.1.1.529 (BA.1).

The first two cases of omicron variant in Romania had been reported on 4 December 2021 in two travelers returning from South Africa, and prior to the moment when our patient was confirmed to be infected with the omicron variant, 10 other cases of omicron infection had been reported in Romania, of which six were in returning travelers from South Africa, leading to a secondary case in one of their household contacts, one was in a returning traveler from Nigeria, and one was in a returning traveler from the UK, with a secondary case in a direct contact.

Since our patient had no history of recent travel outside the country, an extended epidemiological investigation was performed, and all her family members and work contacts were tested by RT-PCR (*n* = 21). All tests came back negative with one exception, that of a co-worker who was identified to be positive for SARS-CoV-2 and sequencing revealed the omicron variant.

This was a 26-year-old female patient (Case 0) who had traveled to the UK from 26 to 30 November 2021, when omicron was starting to be reported in important numbers in the UK [2]. Shortly after returning to Romania, on 2 December 2021, she developed mild symptoms: nasal congestion, headache, otalgia, chills and fatigue. Upon returning to work on 2 December 2021 she performed rapid antigen tests each morning, all results being negative. By 15 December 2021 when she was tested by RT-PCR and confirmed positive as part of the extended epidemiological investigation for her co-worker, her symptoms had mostly subsided, with persistence only of mild cough (Figure 1).

Both patients were young and had no comorbidities or risk factors for severe COVID-19. Both had been vaccinated with Pfizer-BioNTech with the initial two-dose vaccination regimen completed in February 2021 and the third booster dose with Pfizer-BioNTech in October 2021. Patient Case 0 also had a history of a previous episode of COVID-19 in December 2020, prior to vaccination being available in Romania. At that time she had experienced marked symptoms, with chills, important fatigue and dyspnea. On her most recent SARS-CoV-2 anti-spike S1 IgG test, performed in August 2021, Case 0 had an antibody level of 969 BAU/mL on amplified chemiluminescence.

The two patients had had prolonged contact in a closed workspace, both wearing surgical masks, but occasionally taking them off to drink water or to eat indoors. On 16 December 2021 both cases returned to the hospital for a complete biological and imaging investigation. At that time, Case 0 was in the 14th day of illness and Case 1 in the 7th day of illness. Hematological, biochemical and coagulation tests were all within normal ranges for both patients, and neither had developed pneumonia on native chest computed tomography, with peripheral oxygen saturation measured at 99%.

As both patients reported otalgia among presenting symptoms of COVID-19, we also performed an ear–nose–throat (ENT) consult, which revealed nasal congestion and oropharyngeal erythema with normal eardrum in Case 1 and nasal congestion without oropharyngeal erythema with normal eardrum in Case 0.

Evolution was favorable in both cases, both being staged as mild COVID-19, with no occurrence of pneumonia, and full recovery within 10 days, with only persistence of mild unproductive cough for three weeks in Case 0. No further secondary cases were detected among family members and co-workers as part of this investigation.

This undetected transmission of the omicron variant in Romania may not be the first such instance, as reported by other countries [3], but it was certainly the first confirmed case of locally transmitted omicron variant. This highlights the importance of the use of screening for specific mutations and/or viral genome sequencing as part of routine surveillance of the circulating variants in each country, to allow a better understanding of the epidemiology of the pandemic and to inform local measures.

By 31 December 2021, the number of confirmed omicron cases in Romania had reached 92, and the Romanian Ministry of Health confirmed that community transmission of this variant was present in Romania, and that it was anticipated that this would become the dominant variant in the country within the following two weeks [4].

The particularities of the cases we have reported here indicate some of the challenges seen with this novel variant of concern. For previously dominant variants, the widescale use of rapid antigen tests had allowed the expansion of epidemiological surveillance outside of specialized medical centers, to primary care, and to different workplace settings. Rapid antigen tests were also used for screening purposes in educational settings, allowing the safe return to school of pupils and students, and limiting the need for school closures, which have been shown to have an important impact on pupils and parents alike [5]. With a reported reduced sensitivity of rapid antigen tests for detecting the omicron variant [6], also seen in our index case, this may represent a temporary setback in terms of surveillance and, potentially, clinical diagnosis, at least until further information becomes available regarding the diagnostic efficacy of rapid tests, or until newer-generation rapid antigen tests become available.

Furthermore, our cases highlight the importance of routine molecular epidemiological surveillance, not only for targeted variant identification based on the patient’s travel history. The implementation of molecular tests for variant screening may also allow personalized treatment decisions, at least in the beginning of each wave, when variant replacement begins to occur, particularly if variant type can influence the efficacy of different therapeutic options, as is the case with omicron and most first-generation monoclonal anti-spike antibodies [7].

As the pandemic continues to unfold, much remains to be learned and our approach to surveillance, diagnosis and treatment can benefit from timely access to up-to-date molecular testing strategies.

## Figures and Tables

**Figure 1:**
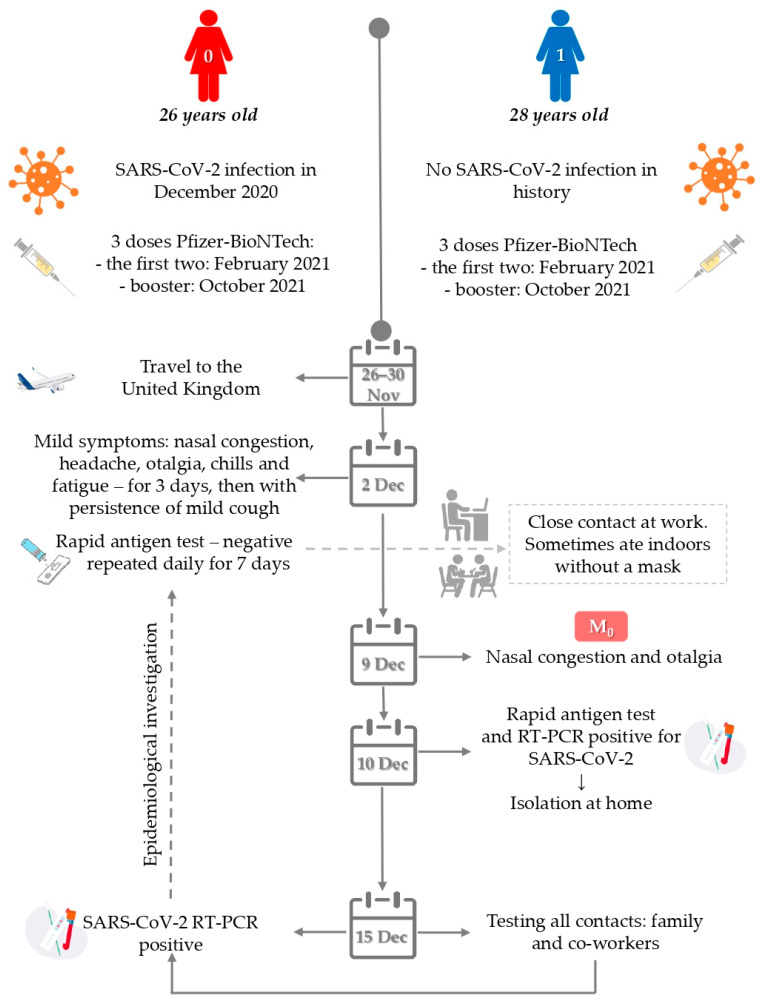
Timeline of epidemiological investigation. M_0_-the trigger for the start of the epidemiological investigation.

## Data Availability

Not applicable.
